# Temporal Trends in Incidence Rates of Lower Extremity Amputation and Associated Risk Factors Among Patients Using Veterans Health Administration Services From 2008 to 2018

**DOI:** 10.1001/jamanetworkopen.2020.33953

**Published:** 2021-01-22

**Authors:** Miao Cai, Yan Xie, Benjamin Bowe, Andrew K. Gibson, Mohamed A. Zayed, Tingting Li, Ziyad Al-Aly

**Affiliations:** 1 Clinical Epidemiology Center, Department of Veterans Affairs, St Louis Health Care Systems, St Louis, Missouri; 2 Veterans Research and Education Foundation of St Louis, St Louis, Missouri; 3 Department of Epidemiology and Biostatistics, College for Public Health and Social Justice, St Louis University, St Louis, Missouri; 4 Section of Vascular Surgery, Department of Surgery, School of Medicine, Washington University in St Louis, St Louis, Missouri; 5 Department of Surgery, Veterans Affairs St Louis Health Care System, St Louis, Missouri; 6 Division of Nephrology, School of Medicine, Washington University in St Louis, St Louis, Missouri; 7 Department of Medicine, School of Medicine, Washington University in St Louis, St Louis, Missouri; 8 Nephrology Section, Medicine Service, Department of Veteran Affairs St Louis Health Care System, St Louis, Missouri; 9 Institute for Public Health, Washington University in St Louis, St Louis, Missouri

## Abstract

**Importance:**

Lower extremity amputation (LEA) is associated with significant morbidity and mortality. However, national temporal trends of LEA incidence rates among US veterans and associated factors have not been well characterized.

**Objective:**

To describe the temporal trends of LEA, characterize associated risk factors, and decompose the associations of these risk factors with changes in temporal trends of LEA among US veterans using Department of Veteran Affairs (VA) services between 2008 and 2018.

**Design, Setting, and Participants:**

This cohort study used VA data from 2008 to 2018 to estimate incidence rates of LEA among veterans using VA services. Cox regression models were used to identify risk factors associated with LEA. Decomposition analyses estimated the associations of changes in prevalence of risk factors with changes in LEA rates. Data were analyzed from October 1, 2007, to September 30, 2018.

**Main Outcomes and Measures:**

Toe, transmetatarsal, below-knee, or above-knee LEA.

**Results:**

A total of 6 493 141 veterans were included (median [interquartile range] age, 64 [54-76] years; 6 060 390 [93.4%] men). Veterans were studied for a median (interquartile range) of 10.9 (5.6-11.0) years. Between 2008 and 2018, rates of LEA increased from 12.89 (95% CI, 12.53-13.25) LEA per 10 000 persons to 18.12 (95% CI, 17.70-18.54) LEA per 10 000 persons, representing a net increase of 5.23 (95% CI, 4.68-5.78) LEA per 10 000 persons. Between 2008 and 2018, toe amputation rates increased by 3.24 (2.89-3.59) amputations per 10 000 persons, accounting for 62.0% of the total increase in LEA rates. Transmetatarsal amputations increased by 1.54 (95% CI, 1.27-1.81) amputations per 10 000 persons; below-knee amputation rates increased by 0.81 (95% CI, 0.56-1.05) amputations per 10 000 persons; and above-knee amputation rates decreased by 0.37 (95% CI, 0.14-0.59) amputations per 10 000 persons. Compared with men, women had decreased risk of any LEA (hazard ratio [HR], 0.34 [95% CI, 0.31-0.37]). Factors associated with increased risk of any LEA included Black race (HR, 1.25 [95% CI, 1.21-1.28]) or another non-White race (ie, Asian, Latino, or other; HR, 2.36 [95% CI, 2.30-2.42]), obesity (HR, 1.59 [95% CI, 1.55-1.63]), diabetes (HR, 6.38 [95% CI, 6.22-6.54]), chronic kidney disease (CKD; eg, CKD stage 5: HR, 3.94 [95% CI, 3.22-4.83]), and smoking status (eg, current smoking: HR, 1.97 [95% CI, 1.92-2.03]). Decomposition analyses suggested that while changes in demographic composition, primarily driven by increased proportion of women veterans, associated with a decrease of 0.18 (95% CI, 0.14-0.22) LEA per 10 000 persons, and decreases in smoking rates, associated with a decrease of 0.88 (95% CI, 0.79-0.97) LEA per 10 000 persons. However, these were overwhelmed by increased rates of diabetes, associated with an increase of 1.86 (95% CI, 1.72-1.99) LEA per 10 000 persons; peripheral arterial disease, associated with an increase of 1.53 (95% CI, 1.41-1.65) LEA per 10 000 persons; CKD, associated with an increase of 1.45 (95% CI, 1.33-1.57) LEA per 10 000 persons; and other clinical factors, including body mass index, cancer, cardiovascular disease, cerebrovascular disease, chronic lung disease, dementia, and hypertension, associated with an increase of 1.45 (95% CI, 1.33-1.57) LEA per 10 000 persons.

**Conclusions and Relevance:**

This cohort study found that incidence rates of LEA among veterans using VA services increased between 2008 and 2018. Efforts aimed at reducing burden of LEA should target the reduction of diabetes, peripheral arterial disease, and CKD at the individual and population levels.

## Key Points

**Question:**

What are the temporal trends of lower extremity amputation (LEA) among US veterans and what risk factors are associated with the changes?

**Findings:**

In this cohort study of 6 493 141 veterans using Veterans Health Administration services, rates of LEA increased between 2008 and 2018. Changes in demographic composition and lower smoking rates were associated with a reduction in LEA incidence rates, but these reductions were more than offset by increased rates of diabetes, peripheral artery disease, and chronic kidney disease.

**Meaning:**

These findings suggest that strategies targeting prevention of diabetes, peripheral artery disease, and chronic kidney disease, as well as further reduction in smoking rates, might contribute to reducing the burden of LEA.

## Teaser

This cohort study examines temporal trends and risk factors associated with lower extremity amputation among veterans using Veterans Health Administration services.

## Introduction

Lower extremity amputation (LEA) is associated with significant morbidity and mortality. Reports on national trends of LEA suggest that among Medicare enrollees, incidence rates of LEA may have declined in the first decade of the 21st century.^1,2,3^ However, changes in demographic factors, due primarily to an aging population, and changes in the prevalence of risk factors (eg, diabetes, peripheral arterial disease [PAD], chronic kidney disease [CKD], and smoking), as well as advances in clinical care may have further altered the prevalence and incidence of LEA in the United States.^1,2,4,5^ Data on recent national trends are not available, and in particular, data on temporal trends of LEA rates among US veterans have not been well characterized.

In this cohort study, we investigate the incidence of LEA among US veterans. The aims of this analysis were to characterize the temporal trends of LEA among users of the Department of Veteran Affairs (VA) health care system over the 11-year period between 2008 and 2018, identify demographic and health characteristics associated with LEA, and decompose the associations of temporal changes in demographic characteristics and other risk factors with changes in rates of LEA between 2008 and 2011.

## Methods

This study was approved by the institutional review board of the VA St Louis Healthcare System. The requirement for informed consent was waived because risk to participants was intangible, per institutional policy. This study is reported following the Strengthening the Reporting of Observational Studies in Epidemiology (STROBE) reporting guideline.

### Data Sources

The VA operates the largest integrated health care system in the United States, the Veterans Health Administration (VHA), with 170 medical centers and 1074 outpatient sites covering more than 9 million veterans.^6^ The VA has also partnered with non-VA health care facilities to provide timely care unavailable within VA facilities under the Veteran Choice Act and the Maintaining Internal Systems and Strengthening Integrated Outside Networks (MISSION) Act.^7^ We used the inpatient, outpatient, and fee basis data sets of the VA Corporate Data Warehouse (CDW) databases to collect *Current Procedural Terminology, Fourth Edition* (*CPT-4*), *International Classification of Diseases, Ninth Revision (ICD-9)*,^8^ and *International Statistical Classification of Diseases and Related Health Problems, Tenth Revision (ICD-10)*^9^ codes.^10,11,12,13,14,15,16,17,18,19,20,21,22,23^ The VA inpatient and outpatient data sets collect data on LEA performed at VA facilities, and the fee basis data set collects data on LEA performed outside of VA facilities but paid for by the VA. The VA Beneficiary Identification Records Locator Subsystem and Vital Status data sets provided patient demographic characteristics (ie, age, sex, and self-reported race) and death dates.^18,21^ We used the VA Managerial Cost Accounting System Laboratory Results to obtain selected inpatient and outpatient laboratory results, including serum creatinine, hemoglobin A_1c_ (HbA_1c_), and albuminuria levels.^20^ The US Renal Data System (USRDS)^24^ was used to identify incidence of kidney transplantation and dialysis. Smoking status was provided by a VA Health Factors data set.^21^ Blood pressure, height, and weight measurements were collected from the CDW Vital Signs domain. The CDW Outpatient Pharmacy domain was used to collect diabetes medication data.

### LEA

LEA was identified using *CPT-4*, *ICD-9 Clinical Modification* (*ICD-9-CM*),^25^ or *ICD-10*^9^ codes (eTable 1 in the Supplement) and further stratified into LEA at toe, transmetatarsal, below knee, or above knee. Incident rate was estimated as the number of new LEAs divided by the number of veterans who had at least 1 encounter in that year.^12,13,14,15,16,26,27,28,29^ When multiple LEA procedure codes occurred within a 30-day period, they were treated as a single LEA to reduce influence on counts from repeated coding resulting from the same procedure or a multistage LEA.

### Clinical Risk Factors

The clinical factors associated with LEA were selected based on previous studies.^30,31,32,33,34,35,36,37,38,39^ Stages of CKD were determined using the mean level of outpatient estimated glomerular filtration rate (eGFR) in that year^40^: eGFR greater than or equal to 60 mL/min/1.73 m^2^ was defined as no CKD, eGFR between 45 and 59 mL/min/1.73 m^2^ was defined as CKD 3A, eGFR between 30 and 44 mL/min/1.73 m^2^ was defined as CKD 3B, and eGFR between 15 and 29 mL/min/1.73 m^2^ was defined as CKD 4. CKD 5 was defined as eGFR less than 15 mL/min/1.73 m^2^ with no history of kidney transplantation or dialysis. Cancer, cerebrovascular disease, cardiovascular disease, dementia, chronic lung disease, and PAD were defined using *ICD-9*^8^ and *ICD-10*^9^ codes (eTable 2 in the Supplement).^41^ Hypertension was defined as median systolic blood pressure greater than or equal to 130 mm Hg or median diastolic blood pressure greater than or equal to 80 mm Hg in 1 year.^42^ Diabetes was defined as any use of antihyperglycemic medications^38,39^ or incidence of HbA_1c_ greater or equal to 6.5% (to convert to proportion of total hemoglobin, multiply by 0.01).^43^ Body mass index (BMI; calculated as weight in kilograms divided by height in meters squared) was calculated from height and weight measurements and further categorized as underweight or weight within reference range (BMI <25), overweight (BMI >25 to <30), or obese (BMI ≥30). Missing BMI values (735 558 veterans [11.3%]) were imputed based on groups of age quintile, race, sex, diabetes, and hypertension status. We used a carry-forward algorithm to define these clinical factors, that is, if a veteran was found to have a clinical factor in an earlier year, they were considered to have that disease in the following years.

### Cohort Construction

#### LEA Incidence Rate Estimation Cohort

For each fiscal year between October 1, 2007, and September 30, 2018, veterans who had at least 1 inpatient or outpatient visit with the VA health care system were considered as the population at-risk in that year. This results in an incidence rate cohort, with approximately 4 million veterans at risk in each of the 11 years (eTable 3 in the Supplement).

#### LEA Risk Profiling Cohort

After presenting the crude and adjusted incidence rates of LEA among veterans and stratified by CKD, diabetes, smoking status, and PAD, we estimated the demographic and clinical risk factors associated with risk of different types of LEA by building a risk profiling cohort of veterans, which is different from the incidence rate cohort (eFigure in the Supplement). The inclusion criteria were the veteran had at least 1 inpatient or outpatient visit or VA enrollment between October 1, 2003, and September 30, 2007; the veteran had no LEA before September 30, 2007 (ie, baseline); and the veteran had been in the VHA system for at least 1 year to ensure capture of relevant clinical diagnosis or laboratory checks. These inclusion criteria resulted in a longitudinal cohort of 6 493 141 veterans. The baseline clinical characteristics, except for CKD, diabetes, and hypertension, were identified using *ICD-9-CM* codes.^25^ Baseline CKD stage was determined using the mean eGFR 1 year before baseline using the aforementioned criteria.

### Statistical Analysis

To characterize the time trend of LEA and 4 major risk factors, we calculated crude rates, as well as age-, sex-, and race-adjusted LEA rates using direct standardization among the incidence rate cohort, with the population structure in 2018 as the reference.^44^ Race was categorized as White, Black, or other race (ie, Asian, Latino, or other). The 95% CIs of the rates and the difference in rates were calculated using normal approximation of binomial CI. We then plotted the rates of LEA and by LEA types, as well as their 95% CIs in each year. To show major differences in LEA rates between cohort groups by primary risk factors, we stratified the rates of LEA by diabetes, CKD category, PAD, and smoking status separately. Since the frequency of kidney transplantation and dialysis were low, we combined them with CKD 5 and labeled them as end-stage kidney disease (ESKD).

To profile the risk factors of LEA, we used Cox proportional hazard models to measure the magnitude of baseline risk factors associated with LEA in the risk profiling cohort of 6 493 141 veterans. Apart from the clinical factors, we also included baseline age, sex, and race as covariates, in which age was included as a natural cubic spline. In Cox regression models, hazard ratios (HRs) and 95% CIs were reported. If the 95% CI of an HR did not include the value 1, the variable was considered statistically significant.

To decompose the factors associated with LEA rate change between 2008 and 2018, we divided risk factors in the previous Cox models into 6 categories: demographic characteristics (ie, age, sex, and race), diabetes, CKD, smoking, PAD, and other clinical factors (ie, BMI, cancer, cardiovascular disease, cerebrovascular disease, chronic lung disease, dementia, and hypertension). In the incidence rate cohort, we calculated the prevalence differences of these factors between 2008 and 2018, estimated the LEA rate differences associated with the change of these factors, and redistributed them to the crude rate change (eAppendix in the Supplement).

All data cleaning and statistical modeling were performed using SAS Enterprise Guide version 7.1 (SAS Institute). The data visualization was performed using R statistical software version 4.0.2 (R Project for Statistical Computing). Data were analyzed from October 1, 2007, to September 30, 2018.

## Results

### Trends of LEA

**Figure 1.**
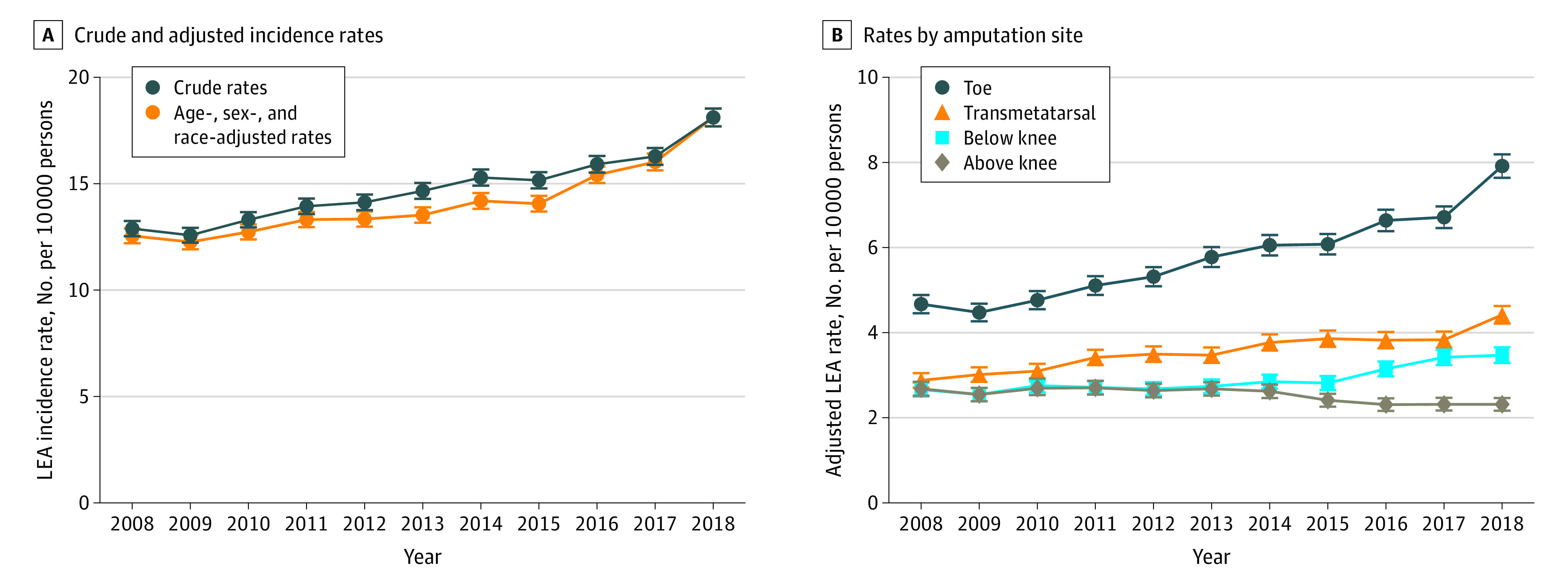
Incidence Rates of Lower Extremity Amputations (LEAs) Among US Veterans

Approximately 4 million veterans received care at the VHA or had non-VHA care that was paid for by the VA each year between 2008 and 2018. The crude rate of LEA increased by 5.23 (95% CI, 4.68-5.78) LEA per 10 000 persons, from 12.89 (95% CI, 12.53-13.25) LEA per 10 000 persons in 2008 to 18.12 (95% CI, 17.70-18.54) LEA per 10 000 persons in 2018 (Figure 1A; eTable 3 in the Supplement). Age-, race-, and sex-adjusted rates showed a similar increase during the same period (Figure 1A).

**Figure 2.**
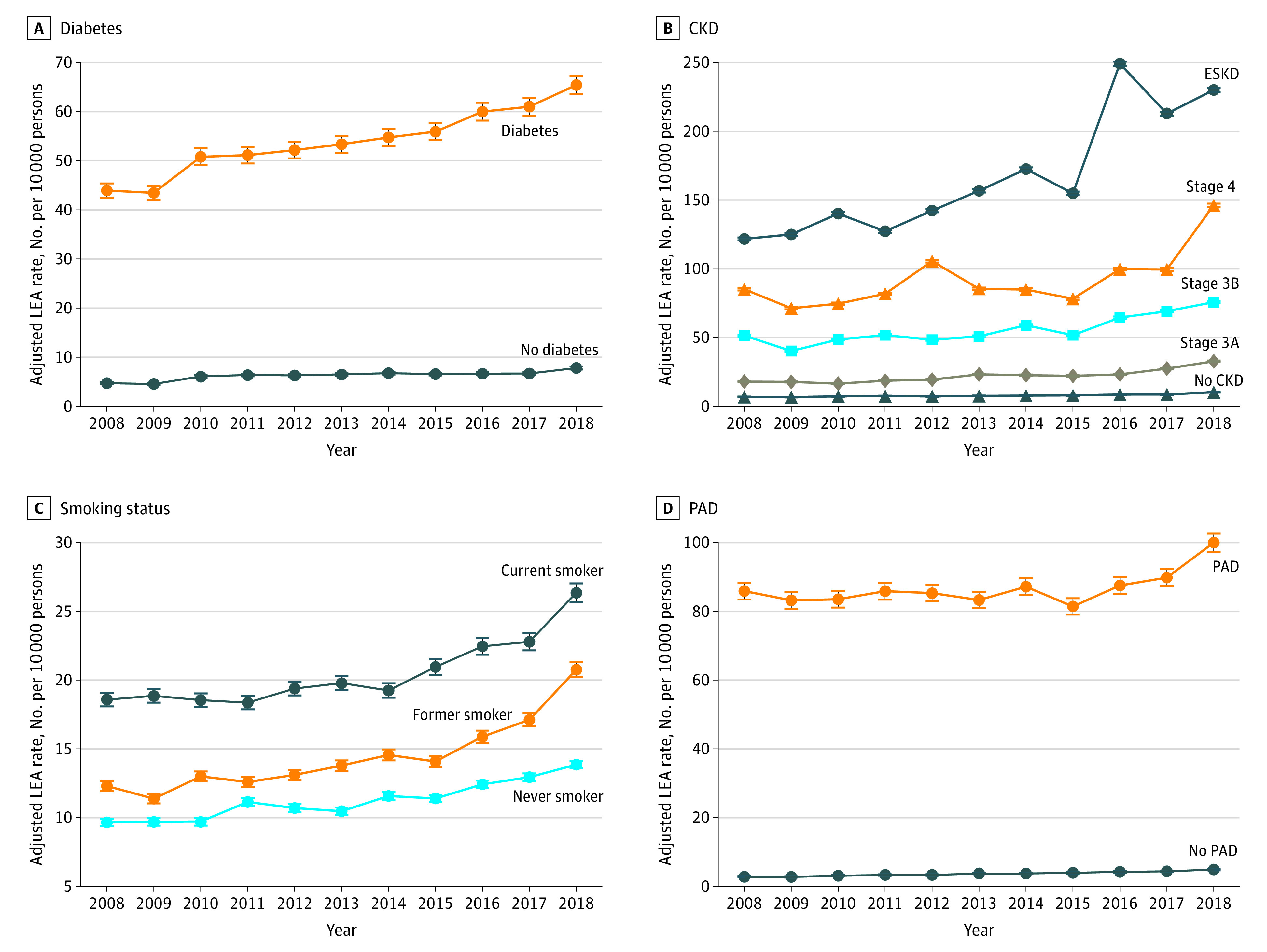
Age-, Sex-, and Race-Adjusted Incidence Rates for Lower Extremity Amputation (LEA)

This increase of LEA between 2008 and 2018 was primarily associated with increases in toe amputations and to a lesser extent modest increases in transmetatarsal and below-knee amputations (Figure 1B; eTable 4 in the Supplement). Crude rates of toe amputations increased by 3.24 (95% CI, 2.89-3.59) amputations per 10 000 persons, accounting for 62.0% of the total increase in LEA during this period (eTable 4 in the Supplement). Crude rates of transmetatarsal amputations increased by 1.54 (95% CI, 1.27-1.81) amputations per 10 000 persons, accounting for 28.9% of the total increase in LEA, and below-knee amputations increased by 0.81 (95% CI, 0.56-1.05) amputations per 10 000 persons, accounting for 15.5% of the total increase in LEA. Crude rates of above-knee amputations decreased by 0.37 (95% CI, 0.14-0.59) amputations per 10 000 persons (eTable 4 in the Supplement). Stratified analyses of age-, race-, and sex-adjusted rates of LEA suggested that rates were higher in veterans with diabetes, CKD, PAD, and active or former smoking status (Figure 2).

### Risk Factors of LEA

In a longitudinal cohort of 6 493 141 participants (median [interquartile range] age, 64 [54-76] years; 6 060 390 [93.4%] men; 5 086 742 [78.4%] White) studied for a median (interquartile range) of 10.9 (5.6-11.0) years, 32 390 participants had an LEA. Demographic and clinical characteristics of the overall cohort and by LEA status are presented in Table 1.

**Table 1.** Baseline Demographic and Health Characteristics of the Overall Cohort and by LEA Status

Characteristic	Overall (n = 6 493 141)	Amputation		*P* value
		No (n = 6 453 904)	Yes (n = 39 237)	
Age, median (IQR), y	64 (54-76)	64 (54-76)	61 (56-68)	<.001
Sex				
Women	432 751 (6.7)	432 030 (6.7)	721 (1.8)	<.001
Men	6 060 390 (93.3)	6 021 874 (93.3)	38516 (98.2)	
Race				
White	5 086 742 (78.4)	5 063 349 (78.5)	23 393 (59.6)	<.001
Black	865 250 (13.3)	858 750 (13.3)	6500 (16.6)	
Other	541 149 (8.3)	531 805 (8.2)	9344 (23.8)	
BMI category				
<25	1 243 067 (19.1)	1 235 769 (19.1)	7298 (18.6)	<.001
25-29.9	3 098 877 (47.7)	3 085 936 (47.8)	12 941 (33.0)	
≥30	2 151 197 (33.1)	2 132 199 (33.0)	18 998 (48.4)	
Diabetes	1 134 278 (17.5)	1 109 831 (17.2)	24 447 (62.3)	<.001
CKD stages				
No CKD	5 255 095 (80.9)	5 228 712 (81.0)	26 383 (67.2)	<.001
CKD 3A	767 363 (11.8)	761 829 (11.8)	5534 (14.1)	
CKD 3B	302 706 (4.7)	300 204 (4.7)	2502 (6.4)	
CKD 4	65 209 (1.0)	64 597 (1.0)	612 (1.6)	
CKD 5	6861 (0.1)	6756 (0.1)	105 (0.3)	
Kidney transplant	1594 (0.0)	1569 (0.0)	25 (0.1)	
ESKD receiving dialysis	94 313 (1.5)	90 237 (1.4)	4076 (10.4)	
Smoking status				
Never	3 246 120 (50.0)	3 234 200 (50.1)	11 920 (30.4)	<.001
Former	1 581 954 (24.4)	1 571 845 (24.4)	10 109 (25.8)	
Current	1 665 067 (25.6)	1 647 859 (25.5)	17 208 (43.9)	
Peripheral arterial disease	423 574 (6.5)	414 596 (6.4)	8978 (22.9)	<.001
Cancer	557 519 (8.6)	553 990 (8.6)	3529 (9.0)	.004
Cardiovascular disease	1 219 971 (18.8)	1 207 827 (18.7)	12 144 (31.0)	<.001
Cerebrovascular disease	422 186 (6.5)	416 841 (6.5)	5345 (13.6)	<.001
Chronic lung disease	749 602 (11.5)	743 401 (11.5)	6201 (15.8)	<.001
Dementia	185 865 (2.9)	184 450 (2.9)	1415 (3.6)	<.001
Hypertension	3 030 809 (46.7)	3 005 746 (46.6)	25 063 (63.9)	<.001

Abbreviations: BMI, body mass index (calculated as weight in kilograms divided by height in meters squared); CKD, chronic kidney disease; ESKD, end stage kidney disease; IQR, interquartile range.

We examined the associations of demographic and clinical characteristics with risks of any type of LEA and toe, transmetatarsal, below-knee, and above-knee amputation using Cox regression models (Table 2). Compared with men, women had decreased risk of any LEA (HR, 0.34 [95% CI, 0.31-0.37]). Other factors associated with increased risk of LEA included Black race (HR, 1.25 [95% CI, 1.21-1.28]), other race (HR, 2.36 [95% CI, 2.30-2.42]), obesity (HR, 1.59 [95% CI, 1.55-1.63]), diabetes (HR, 6.38 [95% CI, 6.22-6.54]), smoking status (current smoker: HR, 1.97 [95% CI, 1.92-2.03]; former smoker: HR, 1.17 [95% CI, 1.14-1.21]), cerebrovascular disease (HR, 1.35 [95% CI, 1.30-1.39]), cardiovascular disease (HR, 1.11 [95% CI, 1.08-1.14]), dementia (HR, 1.22 [95% CI, 1.15-1.29]), hypertension (HR, 1.19 [95% CI, 1.16-1.22]), and PAD (HR, 3.04 [95% CI, 2.95-3.13]). There was a graded association of CKD with risk of amputation in that more advanced stages exhibited higher risk (grade 3A: HR, 1.33 [95% CI, 1.29-1.38]; grade 5: HR, 3.94 [95% CI, 3.22-4.83]) (Table 2). Veterans who had received a kidney transplant had lower risk of amputation (HR, 1.85 [95% CI, 1.20-2.84]) than those with stage 5 CKD and those with ESKD receiving dialysis (HR, 5.58 [95% CI, 5.38-5.79]) (Table 2). The results were consistent for toe, transmetatarsal, below-knee, and above-knee amputations (Table 2).

**Table 2.** Demographic and Health Characteristics Associated With Any LEA and Different Types of LEA

Characteristic	Any LEA	Toe	Transmetatarsal	Below the knee	Above the knee
Age^a^	0.99 (0.99-0.99)	0.98 (0.98-0.98)	0.98 (0.97-0.98)	0.98 (0.98-0.98)	1.01 (1.00-1.01)
Female sex	0.34 (0.31-0.37)	0.33 (0.29-0.38)	0.29 (0.24-0.35)	0.28 (0.24-0.33)	0.40 (0.33-0.47)
Race					
White	1 [Reference]	1 [Reference]	1 [Reference]	1 [Reference]	1 [Reference]
Black	1.25 (1.21-1.28)	1.05 (1.00-1.09)	1.24 (1.16-1.33)	1.38 (1.31-1.45)	1.81 (1.71-1.91)
Other	2.36 (2.30-2.42)	2.35 (2.26-2.43)	2.66 (2.51-2.81)	2.44 (2.34-2.56)	2.71 (2.57-2.85)
Diabetes	6.38 (6.22-6.54)	9.59 (9.24-9.96)	10.39 (1.31-1.48)	6.97 (6.67-7.29)	3.07 (2.92-3.21)
CKD stages					
No CKD	1 [Reference]	1 [Reference]	1 [Reference]	1 [Reference]	1 [Reference]
CKD 3A	1.33 (1.29-1.38)	1.41 (1.35-1.48)	1.41 (1.31-1.52)	1.32 (1.25-1.40)	1.09 (1.02-1.17)
CKD 3B	1.81 (1.73-1.90)	1.96 (1.84-2.09)	2.01 (1.81-2.23)	1.99 (1.84-2.16)	1.41 (1.28-1.55)
CKD 4	2.47 (2.26-2.70)	2.29 (2.02-2.60)	2.53 (2.06-3.10)	2.72 (2.33-3.16)	2.18 (1.85-2.57)
CKD 5	3.94 (3.22-4.83)	4.07 (3.09-5.36)	3.19 (1.95-5.22)	4.56 (3.29-6.33)	3.60 (2.41-5.38)
Kidney transplant	1.85 (1.20-2.84)	1.66 (0.92-3.00)	2.91 (1.45-5.82)	2.01 (1.01-4.02)	0.41 (0.06-2.91)
ESKD receiving dialysis	5.58 (5.38-5.79)	5.05 (4.80-5.32)	6.41 (5.96-6.90)	7.88 (7.46-8.33)	5.84 (5.45-6.26)
Smoking status					
Never	1 [Reference]	1 [Reference]	1 [Reference]	1 [Reference]	1 [Reference]
Current	1.97 (1.92-2.03)	1.66 (1.60-1.72)	1.72 (1.63-1.83)	2.03 (1.94-2.12)	2.95 (2.80-3.12)
Former	1.17 (1.14-1.21)	1.17 (1.12-1.22)	1.21 (1.14-1.29)	1.15 (1.00-1.21)	1.17 (1.10-1.25)
Peripheral arterial disease	3.04 (2.95-3.13)	2.56 (2.46-2.67)	2.75 (2.58-2.93)	3.27 (3.12-3.43)	4.20 (3.98-4.42)
BMI category					
<25	1 [Reference]	1 [Reference]	1 [Reference]	1 [Reference]	1 [Reference]
25-29.9	1.10 (1.06-1.13)	1.14 (1.08-1.19)	1.02 (0.95-1.09)	1.05 (1.00-1.11)	0.99 (0.94-1.05)
≥30	1.59 (1.55-1.63)	1.38 (1.33-1.43)	1.40 (1.30-1.45)	1.57 (1.50-1.64)	1.93 (1.84-2.05)
Cancer	0.97 (0.93-1.00)	0.92 (0.87-0.97)	0.94 (0.86-1.03)	0.92 (0.86-0.99)	1.02 (0.95-1.09)
Cardiovascular disease	1.11 (1.08-1.14)	1.06 (1.02-1.10)	1.05 (0.99-1.12)	1.16 (1.11-1.21)	1.21 (1.15-1.28)
Cerebrovascular disease	1.35 (1.30-1.39)	1.24 (1.18-1.30)	1.23 (1.14-1.33)	1.30 (1.23-1.37)	1.74 (1.64-1.84)
Chronic lung disease	0.96 (0.93-0.99)	0.92 (0.88-0.96)	0.83 (0.77-0.89)	0.92 (0.87-0.97)	1.01 (0.95-1.06)
Dementia	1.22 (1.15-1.29)	1.10 (1.01-1.20)	0.94 (0.80-1.09)	1.01 (0.91-1.12)	1.44 (1.31-1.59)
Hypertension	1.19 (1.16-1.22)	1.17 (1.14-1.21)	1.15 (1.09-1.21)	1.17 (1.13-1.22)	1.18 (1.13-1.24)

Abbreviations: BMI, body mass index (calculated as weight in kilograms divided by height in meters squared); CKD, chronic kidney disease; ESKD, end stage kidney disease; LEA, lower extremity amputation.

^a^ Age was included as restricted cubic spline to account for nonlinearity.

### Factors Associated With LEA Rate Change

**Figure 3.**
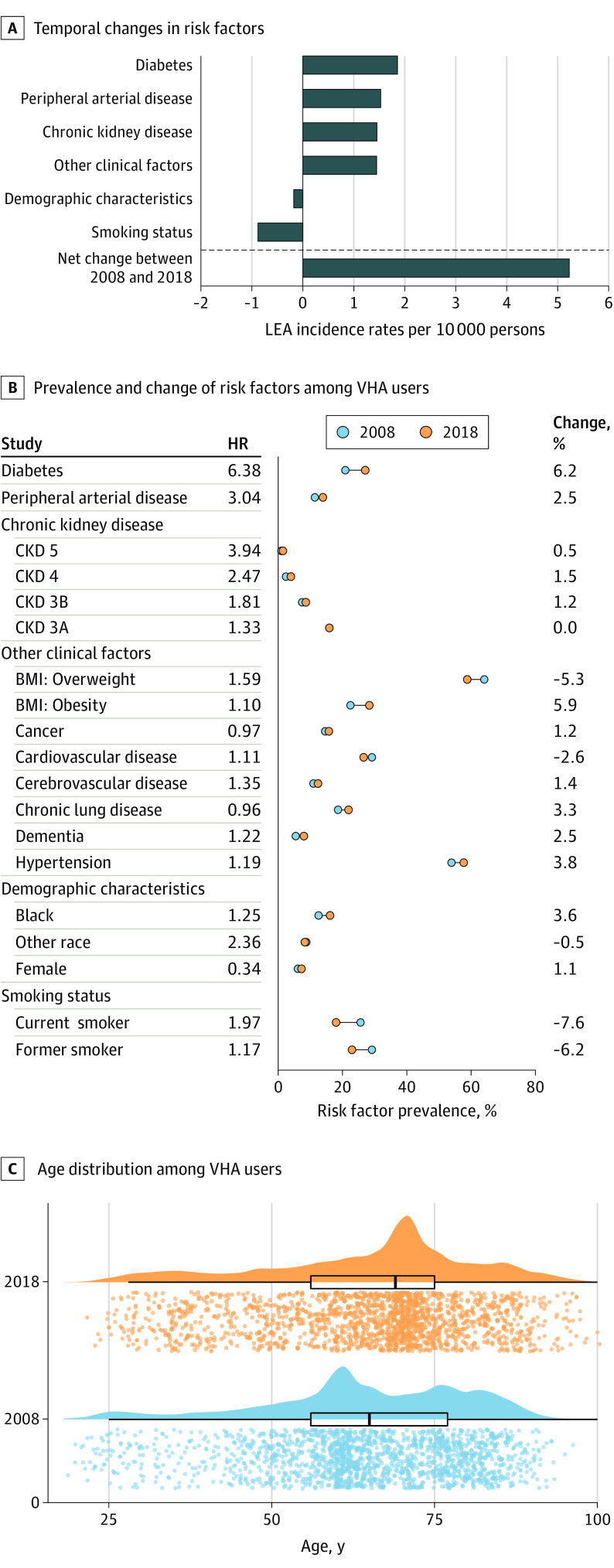
Factors Associated With Change in Rates of Lower Extremity Amputation (LEA) Between 2008 and 2018 BMI indicates body mass index; CKD, chronic kidney disease; and VHA, Veterans Health Administration.

We then evaluated the association of changes in each risk category with the overall change in incidence of LEA between 2008 and 2018 (Figure 3A). While changes in demographic composition were associated with reductions in LEA—primarily driven by an increase in the proportion of women veterans (from 237 820 veterans [6.1%] in 2008 to 286 904 veterans [7.2%] in 2018), which was associated with a decrease of 0.18 (95% CI, 0.14-0.22) LEA per 10 000 persons, and a decrease in smoking rates (current smokers decreased from 999 614 veterans [25.6%] to 713 460 veterans [18.0%]; former smokers decreased from 1 137 902 veterans [29.2%] to 910 272 veterans [22.9%]), which was associated with a decrease of 0.88 (95% CI, 0.79-0.97) LEA per 10 000 persons. These were overwhelmed by increased rates of diabetes (814 769 veterans [20.9%] to 1 073 685 veterans [27.1%]), associated with an increase of 1.86 (95% CI, 1.72-1.99) LEA per 10 000 persons, PAD (444 610 veterans [11.4%] to 551 369 veterans [13.9%]), associated with an increase of 1.53 (95% CI, 1.41-1.65) LEA per 10 000 persons; and CKD (1 040 081 veterans [26.6%] to 1 185 569 veterans [29.9%]), associated with an increase of 1.45 (95% CI, 1.33-1.57) LEA per 10 000 persons. Other clinical factors were associated with ain increase of 1.45 (95% CI, 1.33-1.57) LEA per 10 000 persons. Altogether, these factors were associated with a net increase of 5.23 (95% CI, 4.68-5.78) LEA per 10 000 persons between 2008 and 2018 (Figure 3).

## Discussion

In this large cohort study spanning more than a decade, we describe the temporal trends in LEA among US veterans. The results suggest that between 2008 and 2018, there was an increase in overall LEA rates, which was primarily accounted for by toe amputations and, to a lesser extent, transmetatarsal and below-knee amputations. Rates of above-knee amputations decreased over the same period. Identified risk factors associated with LEA included male sex, Black and other race, obesity, diabetes, smoking, cerebrovascular disease, cardiovascular disease, dementia, hypertension, and PAD. Decomposition analyses suggested that changes in demographic factors and decreased smoking rates were associated with decline in LEA rates, but these salutary trends were more than offset by increases in prevalence of diabetes, PAD, and CKD, which were associated with an overall increase in LEA rates.

The rates for LEA increased over the duration of this analysis, and the 2018 rate was 18.12 (95% CI, 17.70-18.54) LEA per 10 000 persons, an estimate that is higher than those provided by a 2015 analysis of Medicare beneficiaries,^1^ which found that in 2011 LEA rates were approximately 11.9 LEA per 10 000 persons. The differences between these analyses may be a reflection of the underlying differences in age and other risk factors between VA and Medicare beneficiaries and changes that may have occurred over recent years that were not reflected in the 2015 Medicare analysis.^1^ A more recent analysis from 2019 by Geiss and associates^45^ reported a temporal increase in age-adjusted nontraumatic LEA rates in adults with diabetes between 2009 and 2015. That our estimates and those by Geiss et al^45^ suggest a temporal increase in LEA rates is disconcerting and likely reflects the manifestations of the increasing prevalence of amputation risk factors (eg, obesity, diabetes) and calls for a greater attention to halt this increase.

We also observed a divergence in temporal trends with increased rates of toe amputations, which accounted for 62% of the total increase in LEA, and a decrease in rates of above-knee amputations. These divergent trends may not only reflect the increased burden of LEA as a result of increased prevalence of its upstream risk factors, but also the influence of changes in practice patterns, including the implementation of limb salvage strategies and the contributions of VA programs aimed at preventing LEA among veterans.

In our analyses, the demographic and health characteristics associated with LEA, including Black race, diabetes, CKD, smoking, and PAD, are mostly congruent with prior work.^1,3,35,46,47^ Compared with people with no CKD, the risk of LEA exhibited a graded increase with increasing severity of CKD, and it was highest in individuals with ESKD receiving dialysis. Veterans who received kidney transplant exhibited lower risk, likely a reflection of selectivity of kidney transplantation to generally healthier patients.

Our decomposition analyses found a quantitative association of change in risk categories with the overall trend in burden: while demographic changes, such as the introduction of younger returning veterans from recent wars and reduced smoking rates, were associated with a negative change in rates of LEA over the observation period, these were more than offset by increased prevalence of diabetes, PAD, and CKD, which were associated with a net increase in LEA rates. These results suggest that strategies aimed at reducing the burden of diabetes, PAD, and CKD, as well as further amelioration in smoking rates, might yield reduction in the overall burden of LEA.^35^

### Limitations

This study has several limitations. This is an examination of LEA rates among US veterans, and the results may not be generalizable to other populations. While we restricted the analyses to veterans who used VA services (VHA users) and accounted for care provided at VA facilities and care provided outside the VA but paid for by the VA, some VHA users may have received care outside the VA which was paid for by entities other than the VA and may not be accounted for in our data. In our analyses, we relied on administrative data and electronic health records, and although we took care to use validated definitions, misclassification bias may not be completely ruled out. We used definitions for LEA that were optimized to identify site of LEA (eg, toe, transmetatarsal); however, our approach does not distinguish between traumatic and nontraumatic amputations. Our analyses for the examination of risk factors may also be limited by residual confounding.

## Conclusions

In this cohort, we described the trends of LEA, characterized risk factors, and developed decomposition analyses to quantitatively estimate the association of risk categories with overall trends. The results may inform both individual-level and population-level strategies to reduce rates of lower extremity amputations. Overall, our analyses suggest an increasing trend of LEA among veterans between 2008 and 2018. This increase was primarily driven by increases in toe, transmetatarsal, and below-knee amputations. A decline in rates of above-knee amputations was observed. While demographic changes and decreases in smoking rates were associated with a decline in amputation rates, these were more than offset by increased amputation rates associated with diabetes, PAD, CKD, and other risks. Focused attention at the individual and population levels to address risk factors, including smoking, diabetes, PAD, and CKD, may help alleviate the burden of amputation among veterans.

## Notes

Supplement. **eTable 1.** *CPT-4*, *ICD-9-PCS*, and *ICD-10-PCS* Codes for Lower-Extremity Amputation **eTable 2.** *ICD-9* and *ICD-10* Codes for Covariates **eTable 3.** Number of VHA Users, Number of Amputations, and Incidence Rates of Lower-Extremity Amputations in US Veterans Using VHA Services, 2008-2018 **eTable 4.** Number of VHA Users and Incidence Rates of Different Types of Lower-Extremity Amputations in US Veterans Using VHA Services, 2008-2018 **eAppendix.** Decomposition Analyses **eFigure.** Timeline for Cohort Construction
