# Supplementary material for: Temporal Trends in Incidence Rates of Lower Extremity Amputation and Associated Risk Factors Among Patients Using Veterans Health Administration Services From 2008 to 2018
Source: JAMA Netw Open. 2021 Jan 22;4(1):e2033953. doi: 10.1001/jamanetworkopen.2020.33953 (PMC7823225; doi:10.1001/jamanetworkopen.2020.33953)
Supplement: Supplement. — eTable 1. CPT-4, ICD-9-PCS, and ICD-10-PCS Codes for Lower-Extremity Amputation eTable 2. ICD-9 and ICD-10 Codes for Covariates eTable 3. Number of VHA Users, Number of Amputations, and Incidence Rates of Lower-Extremity Amputations in US Veterans Using VHA Services, 2008-2018 eTable 4. Number of VHA Users and Incidence Rates of Different Types of Lower-Extremity Amputations in US Veterans Using VHA Services, 2008-2018 eAppendix. Decomposition Analyses eFigure. Timeline for Cohort Construction [file jamanetwopen-e2033953-s001.pdf]

Supplemental Online Content

Cai M, Xie Y, Bowe B, et al. Temporal trends in incidence rates of lower extremity amputation and associated risk factors among patients using Veterans Health Administration services from 2008 to 2018. *JAMA Netw Open*. 2021;4(1):e2033953. doi:10.1001/jamanetworkopen.2020.33953

eTable 1. *CPT-4*, *ICD-9-PCS*, and *ICD-10-PCS* Codes for Lower-Extremity Amputation

eTable 2. *ICD-9* and *ICD-10* Codes for Covariates

eTable 3. Number of VHA Users, Number of Amputations, and Incidence Rates of Lower-Extremity Amputations in US Veterans Using VHA Services, 2008-2018

eTable 4. Number of VHA Users and Incidence Rates of Different Types of Lower-Extremity Amputations in US Veterans Using VHA Services, 2008-2018

eAppendix. Decomposition Analyses

eFigure. Timeline for Cohort Construction

This supplemental material has been provided by the authors to give readers additional information about their work.

© 2021 Cai M et al. *JAMA Network Open.*

eTable 1. *CPT-4*, *ICD-9-PCS*, and *ICD-10-PCS* Codes for Lower-Extremity Amputation

LEA types	CPT-4	ICD-9-PCS	ICD-10-PCS
Toe	28825	84.11	0Y6P0Z0, 0Y6P0Z1, 0Y6P0Z2, 0Y6P0Z3, 0Y6Q0Z0, 0Y6Q0Z1, 0Y6Q0Z2, 0Y6Q0Z3, 0Y6R0Z0, 0Y6R0Z1, 0Y6R0Z2, 0Y6R0Z3, 0Y6S0Z0, 0Y6S0Z1, 0Y6S0Z2, 0Y6S0Z3, 0Y6T0Z0, 0Y6T0Z1, 0Y6T0Z2, 0Y6T0Z3, 0Y6U0Z0, 0Y6U0Z1, 0Y6U0Z2, 0Y6U0Z3, 0Y6V0Z0, 0Y6V0Z1, 0Y6V0Z2, 0Y6V0Z3, 0Y6W0Z0, 0Y6W0Z1, 0Y6W0Z2, 0Y6W0Z3, 0Y6X0Z0, 0Y6X0Z1, 0Y6X0Z2, 0Y6X0Z3, 0Y6Y0Z0, 0Y6Y0Z1, 0Y6Y0Z2, 0Y6Y0Z3, 0Y6M0Z9, 0Y6M0ZB, 0Y6M0ZC, 0Y6M0ZD, 0Y6M0ZF, 0Y6N0Z9, 0Y6N0ZB, 0Y6N0ZC, 0Y6N0ZD, 0Y6N0ZF
Transmetatarsal	28800, 28805, 28810, 28820	84.12	0Y6M0Z4, 0Y6M0Z5, 0Y6M0Z6, 0Y6M0Z7, 0Y6M0Z8, 0Y6N0Z4, 0Y6N0Z5, 0Y6N0Z6, 0Y6N0Z7, 0Y6N0Z8
below knee	27880,27881, 27888, 27889,	84.13, 84.14, 84.15	0Y6M0Z0, 0Y6N0Z0, 0Y6H0Z1, 0Y6H0Z2, 0Y6H0Z3, 0Y6J0Z1, 0Y6J0Z2, 0Y6J0Z3
above knee	27882, 27884, 27886, 27295, 27590, 27591, 27592, 27596, 27598	84.16, 84.17, 84.18, 84.19	0Y6F0ZZ, 0Y6G0ZZ, 0Y6C0Z1, 0Y6C0Z2, 0Y6C0Z3, 0Y6D0Z1, 0Y6D0Z2, 0Y6D0Z3, 0Y670ZZ, 0Y680ZZ, 0Y620ZZ, 0Y630ZZ, 0Y640ZZ

© 2021 Cai M et al. *JAMA Network Open.*

eTable 2. *ICD-9* and *ICD-10* Codes for Covariates

Comorbidities	ICD-9	ICD-10
Cancer	140.0-172.9, 174.0-208.9	C00.00-C43.99, C45.00-C97.99
Cerebrovascular disease	433.01, 433.11, 433.21, 433.31, 433.81, 433.91	I63.019, I63.119, I63.139, I63.20, I63.219, I63.22, I63.239, I63.59
Cardiovascular Disease	398.91, 402.01, 402.11, 402.91, 410, 411, 413, 414.0, 414.9, 428	I09.81, I11.0, I20.0, I20.1, I20.8, I20.9, I21.09, I21.11, I21.19, I21.29, I21.3, I21.4, I24.0, I24.1, I24.8, I25.10, I25.810, I25.811, I25.812, I25.9, I50.1, I50.20-I50.23, I50.30-I50.33, I50.40-I50.43, I50.9
congestive heart failure	398.91, 402.01, 402.11, 402.91, 404.01, 404.03, 404.11, 404.13, 404.91, 404.93, 425.4–425.9, 428.	I09.9, I11.0, I13.0, I13.2, I25.5, I42.0, I42.5– I42.9, I43.x, I50.x, P29.0
Dementia	290, 294, 331	F01.50, F01.51, F02.80, F02.81, F03.90, F03.91, F04, F05, F06.0, F06.8, G309, G310.1, G310.9, G311, G318.3, G318.4, G318.5, G318.9, G319, G910, G911, G912, G93.7, G94
Hyperlipidemia	272.0, 272.2, 272.4	E78.0, E78.2, E78.4, E78.5
Chronic Lung Disease	491, 492, 493, 496, 518.1, 518.2	J410, J411, J418, J42, J439, J440, J441, J449, J452.0, J452.1, J452.2, J459.01, J459.02, J459.09, J459.90, J459.91, J459.98, J982, J983
Peripheral Artery Disease	440.0-440.9	I70.0, I70.1, I70.2, I70.3, I70.4, I70.5, I70.8, I70.9
Kidney Transplant	V42.0	Z94.0
Dialysis	585.6, V4511, v560, Z568, E87.91	N18.6, Z99.2, Z49.31, Z49.32, Y84.1

© 2021 Cai M et al. *JAMA Network Open.*

eTable 3. Number of VHA Users, Number of Amputations, and Incidence Rates of Lower-Extremity Amputations in US Veterans Using VHA Services, 2008-2018

Fiscal year	Number of VHA users	Number of amputations	Crude incidence rates of amputation per 10,000 persons
2008	3,903,728	5,032	12.89 (12.53-13.25)
2009	3,961,063	4,982	12.58 (12.23-12.93)
2010	4,025,099	5,356	13.31 (12.95-13.66)
2011	4,059,130	5,658	13.94 (13.58-14.30)
2012	4,045,149	5,714	14.13 (13.76-14.49)
2013	4,037,560	5,920	14.66 (14.29-15.04)
2014	4,046,754	6,188	15.29 (14.91-15.67)
2015	4,028,138	6,107	15.16 (14.78-15.54)
2016	3,998,964	6,365	15.92 (15.53-16.31)
2017	3,984,190	6,488	16.28 (15.89-16.68)
2018	3,966,435	7,186	18.12 (17.70-18.54)
Difference between 2018 and 2008		2,154	5.23 (4.68-5.78)

VA, Department of Veteran Affairs; VHA, the Veterans Health Administration.
Fiscal year is from October 1 of the previous calendar year to September 30 of the current calendar year

© 2021 Cai M et al. *JAMA Network Open.*

eTable 4. Number of VHA Users and Incidence Rates of Different Types of Lower-Extremity Amputations in US Veterans Using VHA Services, 2008-2018

Fiscal year	Number of VHA users	Crude incidence rates of amputation per 10,000 persons			
		Toe	Transmetatarsal	Below knee	Above knee
2008	3,903,728	4.67 (4.46-4.88)	2.88 (2.71-3.05)	2.66 (2.50-2.82)	2.68 (2.52-2.84)
2009	3,961,063	4.47 (4.27-4.68)	3.01 (2.84-3.19)	2.54 (2.39-2.70)	2.54 (2.39-2.70)
2010	4,025,099	4.76 (4.55-4.98)	3.10 (2.92-3.27)	2.76 (2.59-2.92)	2.69 (2.53-2.85)
2011	4,059,130	5.11 (4.89-5.33)	3.42 (3.24-3.60)	2.71 (2.55-2.87)	2.70 (2.54-2.86)
2012	4,045,149	5.32 (5.09-5.54)	3.50 (3.31-3.68)	2.68 (2.52-2.84)	2.64 (2.48-2.80)
2013	4,037,560	5.78 (5.54-6.01)	3.47 (3.29-3.65)	2.74 (2.58-2.90)	2.68 (2.52-2.84)
2014	4,046,754	6.05 (5.81-6.29)	3.77 (3.58-3.96)	2.85 (2.68-3.01)	2.62 (2.46-2.78)
2015	4,028,138	6.08 (5.84-6.32)	3.86 (3.67-4.05)	2.82 (2.65-2.98)	2.41 (2.26-2.56)
2016	3,998,964	6.64 (6.38-6.89)	3.82 (3.63-4.02)	3.15 (2.97-3.32)	2.31 (2.16-2.46)
2017	3,984,190	6.71 (6.46-6.97)	3.83 (3.64-4.02)	3.42 (3.24-3.60)	2.32 (2.17-2.47)
2018	3,966,435	7.91 (7.64-8.19)	4.42 (4.21-4.63)	3.47 (3.29-3.65)	2.31 (2.16-2.46)
Difference between 2018 and 2008		3.24 (2.89-3.59)	1.54 (1.27-1.81)	0.81 (0.56-1.05)	-0.37 (-0.59- -0.14)

VA, Department of Veteran Affairs; VHA, the Veterans Health Administration.
Fiscal year is from October 1 of the previous calendar year to September 30 of the current calendar year.

© 2021 Cai M et al. *JAMA Network Open.*

eAppendix. Decomposition Analyses

Let $X_i, i = 1, 2, \ldots, 6$ represent the following six major factors attributing to LEAs among US Veterans.

1. Smoking: including three categories never smoker, former smoker, and current smoker,
2. Demographics: age (categorized as 10-year ranges), gender, and race,
3. Diabetes,
4. Chronic kidney disease (CKD): including no CKD, stage 3A, 3B, 4, and ESKD,
5. Peripheral arterial disease,
6. Other clinical factors: cancer, cerebrovascular disease, cardiovascular disease, dementia, hypertension, chronic lung disease, BMI.

There can be multiple categories for each factor, for example smoking status includes never smoker, former smoker, and current smoker, which is indicated by the second subscript j. So $X_{ij}, i = 1, 2, \ldots, 6; j = 1, 2, \ldots, K_i$ indicates the j-th subcategory for the i-th risk factor. Based on the incident rate cohort, we calculated the prevalence rates of each risk factor subcategory in 2008 ($P_{X_{11}}, P_{X_{12}}, P_{X_{13}}$) and in 2018 ($P^*_{X_{11}}, P^*_{X_{12}}, P^*_{X_{13}}$), as well as the incident rate of LEAs in each risk factor subcategory across the 11 years ($Y_{X_{11}}, Y_{X_{12}}, Y_{X_{13}}$). The contribution of the *i*-th risk factor to the changes of LEA is estimated as:

$$\Delta_i = \sum_{j=1}^{K_i} Y_{X_{ij}} (P^*_{X_{ij}} - P_{ij})$$

This Δ_i is then redistributed to the observed LEA incident rate difference ($D = 5.23$ per 10,000 persons) between 2018 and 2008:

$$\Delta_i^* = \frac{\Delta_i}{\sum \Delta_i} * D$$

Where is Δ_i^* is the final contribution of the *i*-th risk factor to the changes of LEA.

© 2021 Cai M et al. *JAMA Network Open.*

eFigure. Timeline for Cohort Construction

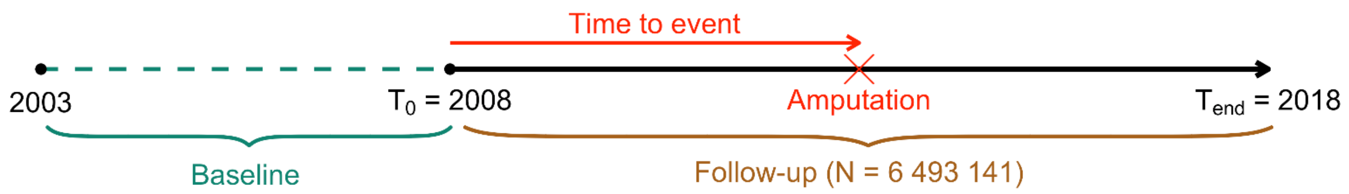

© 2021 Cai M et al. *JAMA Network Open.*
